# The Use of High Frequency Vibration and Clear Aligners in Management of an Adult Patient with Class III Skeletal Malocclusion with Open Bite and Severe Bimaxillary Protrusion: Case Report

**DOI:** 10.3390/dj8030075

**Published:** 2020-07-14

**Authors:** Tarek El-Bialy

**Affiliations:** Division of Orthodontics, Katz Group Centre for Pharmacy and Health Research, School of Dentistry, University of Alberta, Edmonton, AB T6G 2T9, Canada; telbialy@ualberta.ca; Tel.: +1-780-492-2751

**Keywords:** Class III malocclusion, clear aligners, clear aligners, myofunctional therapy

## Abstract

Adult orthodontic patients with skeletal Class III malocclusion, open bite and bimaxillary dentoalveolar protrusion are complex problems that normally require surgical intervention to correct. This is a report of an adult female with a skeletal Class III jaw relationship; in addition, the patient had anterior open bite and bimaxillary dentoalveolar protrusion. The patient also had three premolars in the lower left quadrant. Treatment involved extracting the extra premolar distal to lower left canine, retraction of lower anterior teeth, closing extraction space and anterior open bite utilizing Invisalign clear aligners. The patient initially changed aligners every week before this was changed to 3–5 days after starting to use a high frequency vibration (HFV = 120 Hz) device. Satisfactory results were achieved in a relatively shorter period. Comparing before and after treatment cone beam computed tomography revealed that new bone has been formed labial to the lower incisors after their retraction/correction of their initial proclined position and the use of HFV and myofunctional therapy without gingival graft. The present case report shows the comprehensive multidisciplinary team approach in treatment for such cases and the advantage of using HFV to improve bone formation.

## 1. Introduction

Class III malocclusion is challenging to treat with satisfactory results especially in adults. Many techniques have been reported about the management of these cases with different orthodontic appliances with or without surgical interventions especially in adults. In recent years, clear aligners became more demanded than regular fixed orthodontic appliances due to their acceptability by adults. In addition, clear aligners provide less pain and are more hygienic as well as being more acceptable by adult patients compared to fixed orthodontic appliances. The first reported anterior cross bite case treated by serial clear aligners was in 2009 by Park and Kim [1] that showed serial clear aligners made in the laboratory were able to move teeth sequentially into normal occlusion. A recent study showed that clear aligners are as effective as fixed orthodontic appliances in correcting open bite cases [2]. Recent case reports showed that clear aligners could be successfully used to correct Class III malocclusion and open bite cases with or without surgery. In surgical cases, clear aligners have been successfully used for the correction of the skeletal Class III jaw relationship in adults by surgery first then clear aligners were used to finish the patient’s occlusion [3,4,5]. Extraction of lower incisor also has long been reported to be an acceptable treatment approach in Class III malocclusion cases especially in adults [6,7,8]. The use of high frequency vibration (HFV) has been introduced in orthodontics and recent reports showed that it does not only accelerate tooth movement, but also it increases bone density at the end of active treatment in human as well as after extraction in rats [9,10,11,12,13,14,15]. The anabolic effect of HFV during orthodontic treatment makes it unique especially in cases with gingival recession or compromised crown/root ratio. The increased osteoblastic activity of HFV is reported due to its upregulation of osteogenic transcription factors (RUNX2, Foxo1, Osterix and Wnt signaling factors) [13]. In cases with severe proclination of lower incisors, loss of alveolar bone can be seen labial to the proclined incisors that may also show gingival recession [16,17,18,19]. Interdisciplinary treatment plan including orthodontics and myofunctional therapy have shown successful results in similar cases [20]. This case report show the utilization of HFV and myofunctional therapy to facilitate orthodontic treatment of an adult with skeletal Class III jaw relationship with anterior open bite and bimaxillary protrusion in addition to gum recession labial to lower incisor without the need for gingival graft. 

## 2. Materials and Methods

### 2.1. Case Presentation 

**Figure 1:**
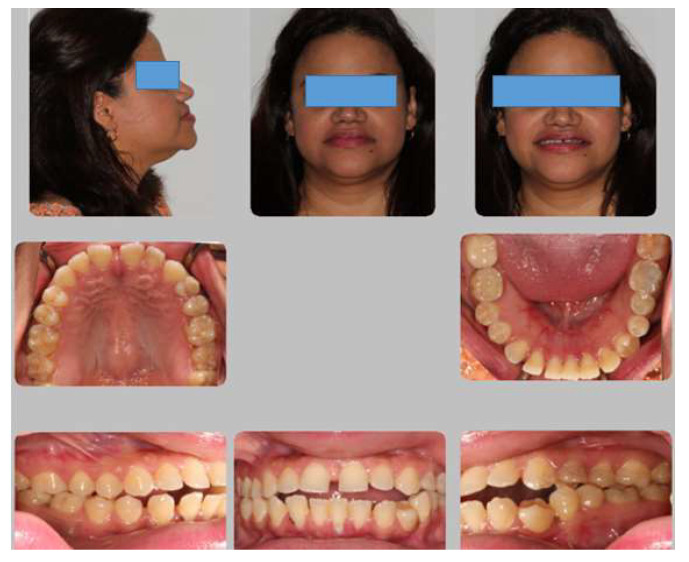
Initial clinical records showing anterior open bite with bi-maxillary protrusion with extra third premolar distal to lower left canine.

**Figure 2:**
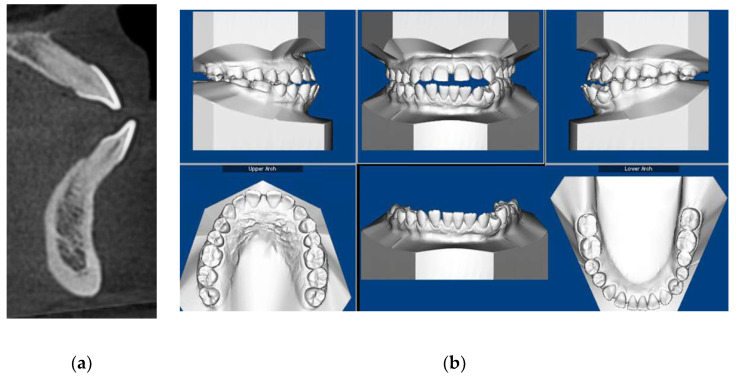
(**a**) Cone-beam computed tomographs (CBCT)-driven sagittal screen of lower incisor showing its severe proclination with no bone appears on most of the labial surface of the root; (**b**) Initial digital models.

This forty-five year old female presented with chief concerns that she wanted to close her anterior open bite and close spaces between anterior teeth as well as move her protruded front teeth lingually and correct her under bite. Initial clinical records (Figure 1 and Figure 2) show concave profile with prominent chin projection and competent lips. She reported that she breaths through her nose but she had an anterior tongue thrusting habit. Intraoral examination revealed that the patient had an extra (third) premolar in the lower left quadrant of her mouth. Right molars and canine relationships revealed half cusp Class III occlusion and left side molars and canine relationships were Class I occlusion (Figure 1 and Figure 2b). The patient also presented with anterior and lateral cross bites that were extended from upper right to upper left first premolars with a 3-mm open bite. In addition, the patient had upper 5-mm and lower 12-mm spacing (considering the spaces of the lower left extra (third) premolar). Bolton analysis revealed total mandibular excess of 0.72 mm (total ratio 92.02%) and mandibular anterior excess of 1.04 mm (anterior ratio of 79.39%). Upper dental midline was centered to the patient’s facial midline and lower dental midline was deviated to the left by 2 mm from the upper dental midline. The patient gave her informed consent for inclusion before they participated in the study. The study was conducted in accordance with the Declaration of Helsinki, and the protocol was approved by the Ethics Committee of the University of Alberta (Project identification code: Pro00074117, date of approval: 13 December 2017).

**Figure 3:**
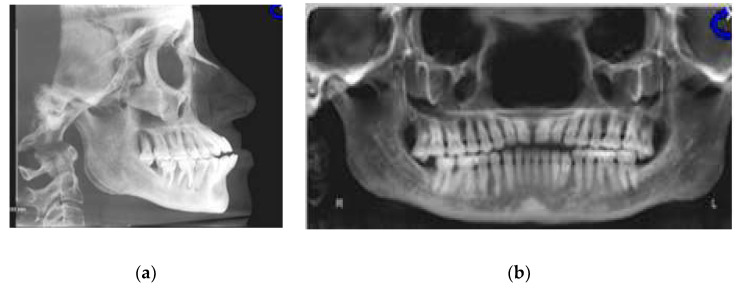
(**a**) CBCT-driven cephalometric radiograph showing anterior cross bite and open bite as well as bimaxillary protrusion; (**b**) CBCT-driven panoramic radiograph showing extra premolar distal to lower left canine and missing lower third molars as well as over erupted upper third molars.

**Figure 4:**
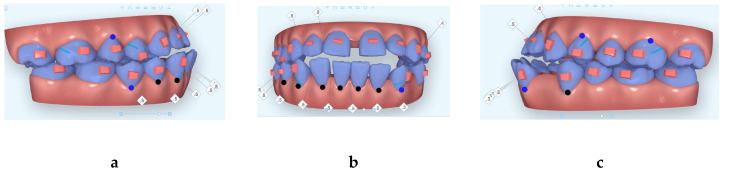
Images of the beginning of first Clincheck (**a**): right side; (**b**) frontal view and (**c**) left side.

Initial and final cone-beam computed tomographs (CBCT) were obtained using the i-CAT FLX (Imaging Sciences International, Hatfield, PA) with a scanning time of 3.7 s, 18.54 mA, 120 kVp, with a field of view of 23 cm × 17 cm and slice thickness of 0.3 mm. All CBCT images were taken by one trained and calibrated examiner using standardized imaging techniques. Cephalometric radiographs and analysis (Figure 3a, Table 1) revealed a Class III apical base relationship (ANB = −0.5°) with both maxillary and mandibular apical base prognathism relative to the anterior cranial base. Upper incisors were proclined relative to SN (119°) and protruded relative to NA (7.5 mm). Lower incisors were severely proclined relative to mandibular plane (106.3°) and protruded relative to NB (9.4 mm). Together with lower incisors forward position, there was lack of labial bone relative to lower incisors (Figure 2a) and 4-mm gingival recession labial to lower left central incisor. However, there was no other deep periodontal pocket or other periodontal concern. The patient also had lower third molars missing and super erupted upper third molars (Figure 3b).

### 2.2. Treatment Plan and Progress

**Figure 5:**
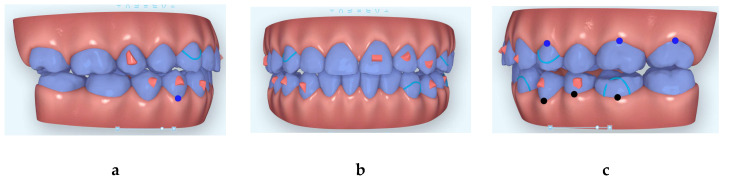
Images of the beginning of second Clincheck (**a**): right side; (**b**) frontal view and (**c**) left side.

**Figure 6:**
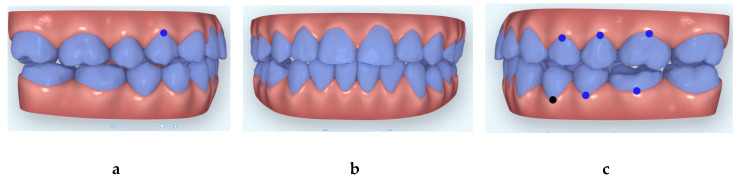
Images of the beginning of third Clincheck (**a**): right side; (**b**) frontal view and (**c**) left side.

**Figure 7:**
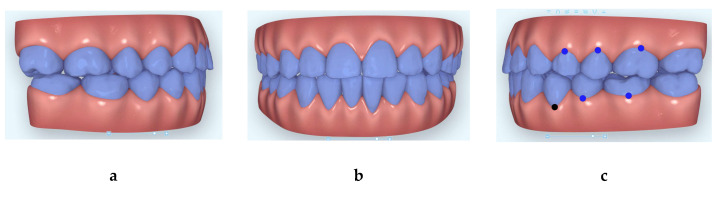
Images of the beginning of final Clincheck (**a**): right side; (**b**) frontal view and (**c**) left side.

Different treatment options were discussed with the patient including orthognathic surgery to reposition upper and lower jaws relative to the anterior cranial base and periodontal gingival graft to cover lower incisors gingival recession in addition to orthodontic treatment with either fixed orthodontic appliance or clear aligners. However, the patient declined the surgical options and accepted clear aligners orthodontic option. It was important to remove the third (extra) premolar distal to lower left canine and also the removal of the over erupted upper third molars as they had no opposing teeth. The patient was referred to a periodontist for periodontal consultation regarding her periodontal status and consultation for gum graft labial to lower incisors; however, the patient declined periodontal surgery. Initial digital treatment planning (Clincheck) instructions were to virtually remove 3.4 (lower left first (buccally tilted extra-premolar)) premolar, to distalize 3.3 into the extraction space after virtually removing the extra-premolar and to move lower incisors posteriorly and to move lower midline to patient’s left side to match upper midline. Additionally, instruction was to avoid mesializing the lower left buccal segment, maintain initial molar relationship but improve their occlusion. In addition, IPR was prescribed between 3.2 and 3.1 and between 4.1 and 4.2 to minimize black triangle and to manage lower mandibular Bolton excess. Anchorage consideration during anterior teeth retraction was the utilization of horizontal extrusion attachments on the mesial parts of all molars to provide crown tip back and counter root moment in the mesial direction to counteract the mesial reaction force on the posterior teeth during anterior teeth retraction. Initial set of aligners included 35 aligners (Figure 4) to move lower left canine to the extraction space of the extra (third) premolar and to help with lower midline correction. The patient was instructed to wear the aligners full time and change them only when the new aligners would fit her teeth without too much pressure felt by the patient. Myofunctional therapy exercises provided to the patient in verbal and written format to bite on her back teeth during swallowing and to keep her tongue in the palate where her tip of the tongue to touch the incisive papilla. The patient managed to change her aligners every 7–10 days. At aligner 22/35 of the first set of aligners, lower left canine was not tracking very well, so a new digital scan was obtained for additional aligners (27 more aligners) (Figure 5)**.** Upon insertion of the new aligners set, the patient was given the HFV (Vpro5, Propel, NY, USA) device to ensure best fit or best aligners seating to minimize possible future non tracking of teeth and the patient was instructed to use this for 5 min per day to help in accelerating the tooth movement and to help with aligners seating to ensure that teeth track very well into the aligners. The patient reported that she could change her aligners every 3–5 days without too much pressure felt from the new aligners when she used the Vpro5 for 5 min every day. Two more additional aligners sets (32 more aligners) (Figure 6 and Figure 7) sets were ordered for fine-tuning the occlusion for a total of 94 aligners. Since the second set of aligners, the patient changed her aligners on average every 4 days. Patient was given four sets of Invisalign Vivera retainers to use for full time for a year and nighttime forever. It is important to mention that the patient had to go through extensive myofunctional therapy (instructions) to retrain the tongue to stay in the patient’s palate to avoid relapse.

## 3. Results

**Figure 8:**
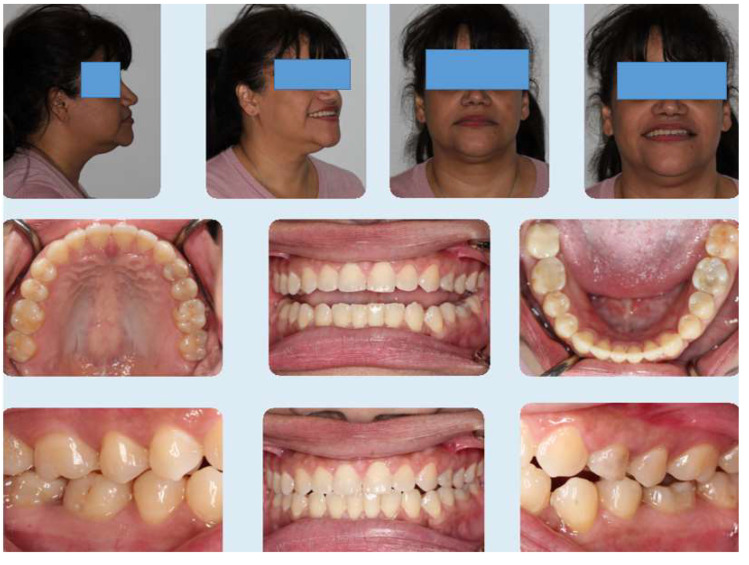
Final photos showing improvement in the patient’s initial chief complaints including open bite and spacing as well as cross bite.

**Figure 9:**
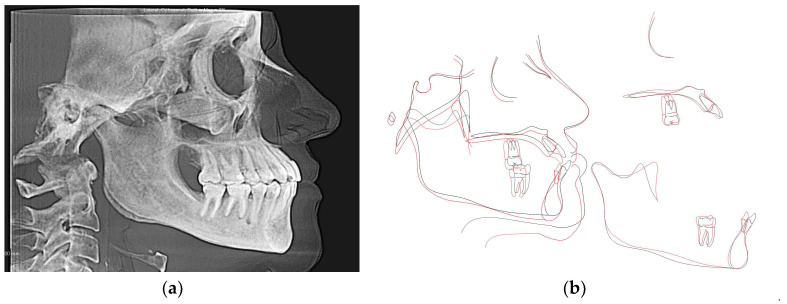
(**a**) Final CBCT-driven cephalometric radiograph and (**b**) cephalometric superimposition of before (black) and after (red) treatment cephalometric tracings showing improvement in the patient’s initial chief complaints including open bite and spacing as well as crossbite.

**Figure 10:**
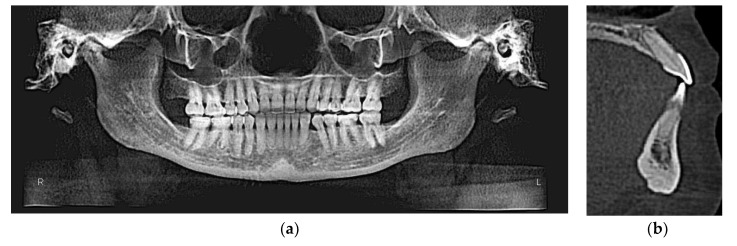
(**a**) Final CBCT-driven panoramic radiograph showing complete retraction of lower left canine; (**b**) final CBCT-driven sagittal screen showing bone formation labial to lower incisors compared to initial one with severely proclined lower incisor.

Patient completed her active treatment over 15 months and final records (Figure 8, Figure 9 and Figure 10) show acceptable occlusion relative to her initial malocclusion. 

### 3.1. Clinical Records

The patient had no gingival graft done and final photos (Figure 8) show very good covering of the lower incisors by maintaining good oral hygiene and retraction of lower incisors into normal position compared to initial photos. Anterior cross bite and spacing have been corrected.

### 3.2. CBCT-Driven Radiographs

#### 3.2.1. Cephalometric Radiographs and Analysis 

Final cephalometric analysis shows improvement in upper and lower incisors inclinations and protrusion relative to apical bases compared to the initial values (Table 1, Figure 9a,b).

#### 3.2.2. Panoramic and Sagittal Screen Radiographs 

Figure 10a,b show the final CBCT-driven panoramic radiograph; the extraction space of the third premolars has been closed by bodily retraction of lower left canine and lower incisors as well. Additionally, it can be noted that upper third molars have been removed during the course of treatment. Final CBCT-driven sagittal screen of lower incisor (Figure 10b) shows more bone labial to lower incisor compared to the initial similar sagittal screen (2a). 

## 4. Discussion

Treatment of open bite using the extraction of teeth utilizing clear aligners has been recently reported [2,3,5]; however, the use of HFV in conjunction with myofunctional therapy without gingival graft is the most important and new finding in this case report. The important observation to report here is the new bone formation labial to the initially proclined lower incisors after using HFV without gingival graft to cover the gum recession labial to the affected lower incisor (Figure 6 b). It has been reported before that HFV not only can accelerate tooth movement with clear aligners [10,11,12] but also it increase bone formation and density after orthodontic treatment and after extraction of teeth as well [12,13,14,15,16]. Previous reports have shown that the increased bone density effect of HFV is mediated through its upregulation of osteogenic transcription factors (RUNX2, Foxo1, Osterix and Wnt signaling factors) [13]. The biological mechanism of accelerating tooth movement by HFV has been reported in detailed before [16]. This supports the reported findings in this case report that new bone formation has been observed labial to lower incisors after orthodontic treatment and the use of HFV. This also supports the hypothesis that the increased bone density by HFV can be helpful during the retention period [12], though this needs to be studied in detail in future studies. Although orthodontic labial movement of lower incisors may not be a risk factor in inducing gingival recession [17], the reverse could be true that moving lower incisors lingually after being proclined can help with the use of HFV. The multidisciplinary approach done in this case report that myofunctional therapy to rehabilitate the tongue in severely proclined lower incisors can be corrected by moving lower incisors to their normal position using clear aligners without free gingival graft [18,19,20]. It is important to consider the use of HFV in similar cases in the future so as to help bone formation in areas with previous gingival recession. The utilization of myofunctional therapy to rehabilitate the tongue from anterior tongue thrusting habit is extremely important to implement to assure long-term stability of the achieved results [21]. Some limitations of the results in this case report included root parallelism of lower left first premolar and lower canine as well as maximum interdigitation in this area; however, the patient was satisfied and did not want to proceed with any further occlusal detailing. Moreover, another limitation is that if finishing the case without deep overbite to consider, possible future relapse may occur in the open bite correction. 

## 5. Conclusions

1. The use of multidisciplinary approach in treating similar cases of severe open bite and the coordination between the orthodontist and myofunctional therapist is extremely important to ensure timely treatment and long-term stability.
2. The use of HFV can be an important treatment adjunctive therapy in similar cases to minimize orthodontic treatment time as well as to help bone formation where gingival recession was initially present due to the severely proclined incisors.

## Figures and Tables

**Table 1:** Cephalometric analysis of before and after treatment.

Measurement	Initial	Final	Norm
SNA (°)	86.2	86.3	82
SNB (°)	86.7	86	80.9
SN - MP (°)	28.9	29.8	32.9
FMA (MP-FH) (°)	20.8	18.9	23.9
ANB (°)	−0.5	0.3	1.6
U1 - NA (mm)	7.5	4.8	4.3
U1 - SN (°)	119	109.9	102.8
L1 - NB (mm)	9.4	3.7	4
L1 - MP (°)	106.3	87.4	95
Lower Lip to E-Plane (mm)	0.5	−2.8	−2
Upper Lip to E-Plane (mm)	−6.8	−6	−6
